# Cell Patterning Technology on Polymethyl Methacrylate through Controlled Physicochemical and Biochemical Functionalization

**DOI:** 10.3390/bios13100904

**Published:** 2023-09-23

**Authors:** Enrique Azuaje-Hualde, Job Komen, Juncal A. Alonso-Cabrera, Albert van den Berg, Marian M. de Pancorbo, Andries D. van der Meer, Fernando Benito-Lopez, Lourdes Basabe-Desmonts

**Affiliations:** 1 Microfluidics Cluster UPV/EHU, BIOMICs Microfluidics Group, Lascaray Research Center, University of the Basque Country UPV/EHU, 01006 Vitoria-Gasteiz, Spain; enrique.azuaje@ehu.eus (E.A.-H.); juncalanne.alonso@ehu.eus (J.A.A.-C.); 2 Bioaraba Health Research Institute, Microfluidics Cluster UPV/EHU, 01009 Vitoria-Gasteiz, Spain; 3 BIOS Lab on a Chip Group, MESA+ Institute for Nanotechnology, University of Twente, P.O. Box 217, 7500 AE Enschede, The Netherlands; jobkomen@gmail.com (J.K.); a.vandenberg@utwente.nl (A.v.d.B.); 4 Microfluidics Cluster UPV/EHU, Analytical Microsystems & Materials for Lab-on-a-Chip (AMMa-LOAC) Group, Analytical Chemistry Department, University of the Basque Country UPV/EHU, 48940 Leioa, Spain; 5 BIOMICs Research Group, Lascaray Research Center, University of the Basque Country UPV/EHU, 01006 Vitoria-Gasteiz, Spain; marianpancorbo@gmail.com; 6 Applied Stem Cell Technologies, TechMed Centre, University of Twente, P.O. Box 217, 7500 AE Enschede, The Netherlands; andries.vandermeer@utwente.nl; 7 Basque Foundation of Science, IKERBASQUE, María Díaz Haroko Kalea, 3, 48013 Bilbao, Spain

**Keywords:** polymethyl methacrylate, cell patterning, microcontact printing, microfluidic device, commercialization, cell-based microsystems

## Abstract

In recent years, innovative cell-based biosensing systems have been developed, showing impact in healthcare and life science research. Now, there is a need to design mass-production processes to enable their commercialization and reach society. However, current protocols for their fabrication employ materials that are not optimal for industrial production, and their preparation requires several chemical coating steps, resulting in cumbersome protocols. We have developed a simplified two-step method for generating controlled cell patterns on PMMA, a durable and transparent material frequently employed in the mass manufacturing of microfluidic devices. It involves air plasma and microcontact printing. This approach allows the formation of well-defined cell arrays on PMMA without the need for blocking agents to define the patterns. Patterns of various adherent cell types in dozens of individual cell cultures, allowing the regulation of cell–material and cell–cell interactions, were developed. These cell patterns were integrated into a microfluidic device, and their viability for more than 20 h under controlled flow conditions was demonstrated. This work demonstrated the potential to adapt polymeric cytophobic materials to simple fabrication protocols of cell-based microsystems, leveraging the possibilities for commercialization.

## 1. Introduction

In recent years, the use of new microfabrication techniques and the development of microfluidic devices have had an impact on a wide range of research areas. This trend can be attributed to the reduction in the production cost, the reduction in the use of reagents, the simplification of methods, the portability of devices and the adaptation to user-friendly interfaces. In addition, microfluidics improve process performance compared to conventional methods [1]. In the field of biological and biomedical research, microtechnologies for cell monitoring and biosensing are being developed with the aim of enhancing control over various cellular interactions, enabling more precise analysis of cellular behaviors [2,3]. In particular, the generation of cell patterns, where arrays of hundreds of single cellular replicates with controlled localization, distribution and cell–cell contact can be obtained, have demonstrated the capability to achieve high-throughput, non-invasive and real-time optical analysis of cell behaviors. However, most of the cell monitoring and biosensing platforms developed in research laboratories rely on conventional cell culture materials, such as glass, which are not truly scalable to industrial mass-production processes. This greatly limits the commercialization potential of these microtechnologies. To create new commercial tools for the pharmaceutical and biomedical industries, it is desirable to extend the manufacturability of these novel microtechnologies to plastic substrates, which are less fragile and offer a wider range of optical properties [4,5].

Polymethyl methacrylate (PMMA) is a material commonly employed in the fabrication of microfluidic devices due to its durability, ease of manipulation and cost-effectiveness. Considering these factors, PMMA provides a versatile option for the design of a diverse range of microfluidic devices [6,7]. However, while PMMA presents moderate to good biocompatibility, it has low cell adhesion properties and has been described as bioinert and cytophobic [8,9,10]. This phenomenon can be attributed to the innate low wettability of the material and the absence of functional chemical cues that are necessary for the binding of biomolecules, thus impeding the deposition and formation of microenvironment components that are essential for optimal cell adhesion and maintenance [11,12,13]. This limitation severely reduces its potential use for the generation of cell monitoring microfluidic devices.

In order to address PMMA’s limitations, a variety of methods have been investigated in order to enhance cell adhesion on the material by increasing its surface hydrophilicity, including but not limited to chemical- and light-based treatments of the polymer [14,15,16,17]. However, one of the most facile and quick techniques to augment the surface hydrophilicity of a polymer is via air or oxygen plasma treatment. In essence, the free radicals produced within a plasma chamber can catalyze the oxidation of the surface of a material, thereby exposing polar functional groups that improve the hydrophilicity of the surface. The use of plasma treatment has been shown to enhance the hydrophilicity of PMMA surfaces, leading to augmented interactions between cells and proteins and the material. This enables the adhesion of extracellular matrix proteins, providing better adhesion of cells to PMMA surfaces [15,18,19,20].

In order to generate arrays of discrete cell groups on a single sample, microcontact printing (µCP) of proteins, an easy and fast method that requires minimum equipment or expertise, emerges as a powerful choice that can be used within any laboratory. The method, which results in the production of patterned surfaces with contrast between adherent and non-adherent areas, consists of the dry transfer of cell adhesion proteins from a polydimethylsiloxane (PDMS) stamp to a substrate, replicating the features carved on it [21,22]. Only a small number of studies are reported in the literature that have utilized a combination of plasma treatment and microcontact printing (µCP) of proteins to improve cell adhesion on PMMA surfaces. Schmalenberg et al. and Wang et al. have reported successful applications of combined plasma treatment of PMMA surfaces and µCP of laminin for the adhesion and growth of Schwann cells and nerve cells, with high potential to function as nerve guides and promote nerve regeneration [23,24,25]. Recently, Hager et al. studied subcellular patterning of different proteins through µCP on plasma-activated and epoxy-functionalized polymeric surfaces, including PMMA, and demonstrated that PMMA could be a feasible and flexible alternative to conventional cell culture materials, like glass, for µCP, presenting comparable optical properties [5]. Although there is growing interest in using these polymeric materials as substitutes for traditional cell culture materials in the development of novel cell monitoring microtechnologies, there has been limited exploration of the combination of physicochemical and biochemical modifications of PMMA surfaces to control cell adhesion on the typically cytophobic material.

**Figure 1:**
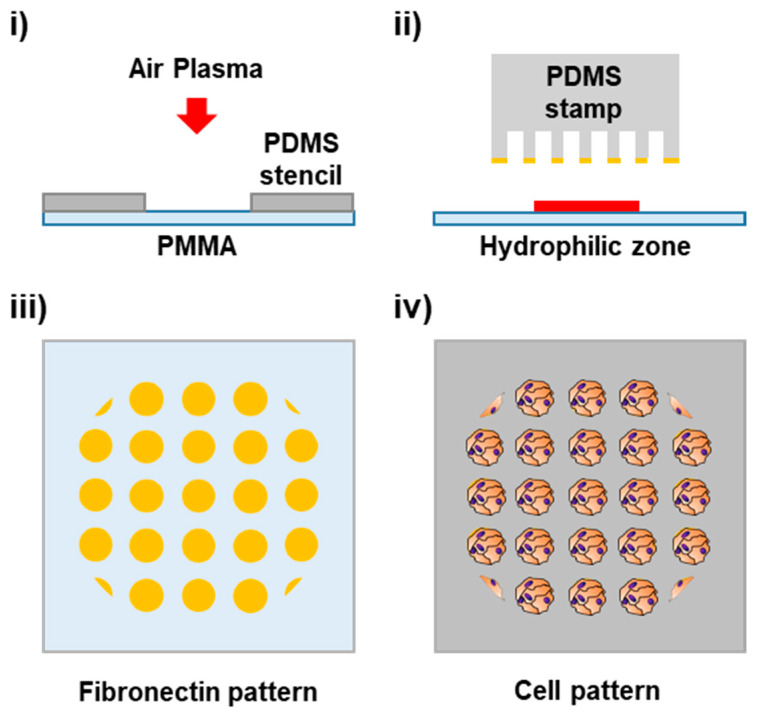
Cartoon of cell patterning process on PMMA. (**i**) Generation of a hydrophilic zone on the PMMA surface through localized plasma treatment using a PDMS stencil. (**ii**) Microcontact printing of cell adhesion proteins. (**iii**) Pattern of a fibronectin dot array. (**iv**) Cell isles pattern.

In this work, a method that combines plasma treatment and µCP was evaluated for the controlled patterning of cells on PMMA, in order to achieve functional and controlled cell adhesion on the surface of cytophobic polymeric materials. Air plasma treatment was utilized in order to enhance hydrophilicity on the PMMA surfaces, using PDMS slabs as stencils for the generation of hydrophilic zones with high contrast to their hydrophobic surroundings. µCP of cell adhesion proteins was directly applied to promote the adhesion of various cell types on dozens to hundreds of pre-defined architectures with controlled cell–cell and cell–material interactions. The cell patterns were integrated in a simple microfluidic device as a proof of concept, demonstrating the direct applicability of this methodology for an optical and rapid monitoring and analysis of patterned live cells inside a microfluidic device (Figure 1).

## 2. Materials and Methods

### 2.1. Materials

Primary human hair follicle-derived mesenchymal stromal cells (hHF-MSCs) were obtained from human follicles (passages 6 to 9). Prostate cancer cells PC3 and colorectal cancer cells HCT116 were purchased from ATCC, Manassas, VA, USA. Bovine plasma fibronectin, Dulbecco’s modified eagle’s medium (DMEM), F-12 Medium, fetal bovine serum (FBS) and penicillin/streptomycin (P/S) were purchased from Fisher Scientific, Spain. Bovine serum albumin was purchased from Sigma Aldrich, Madrid, Spain. Polydimethylsiloxane silicone (PDMS) elastomer and curing agent SYLGARD 184 were purchased from Ellsworth adhesives, Madrid, Spain. PMMA Plexiglas 4 mm was purchased from Evonik Industries AG, Essen, Germany. Pressure-sensitive adhesive (PSA) ARcare 8939 was purchased from Adhesive Research, Limerick, Ireland. Luers and reservoirs (male 1/16 in barb and female Luer Lok compatible connectors white base) were purchased from Microfluidics ChipShop, Jena, Germany. Tubing was purchased from Altmann Analytik GmbH & Co, Munich, Germany.

### 2.2. Selective Oxidation of PMMA Surface

In order to generate controlled hydrophilic zones within PMMA surfaces, air plasma treatment was used for the oxidation of the surfaces. PDMS slabs were applied as stencils for non-treated areas. In summary, 3 mm holes were punched on flat PDMS slabs (elastomer and curing agent proportion 10:1, 0.8 cm height). After carefully washing with ethanol, the PDMS slabs were put in close contact with PMMA slides (3 cm × 5 cm × 0.4 cm, length × width × height). The PDMS-protected PMMA was oxidized using air plasma inside of a plasma cleaner (BlackHole, Paris, France) with a power of 29.6 W for 150 s.

To verify the generation of controlled hydrophilic zones, the spreading of a distilled water (DI water) drop was analyzed. An amount of 1 µL of DI water was deposited on untreated PMMA, plasma-treated PMMA and PDMS-protected PMMA surfaces after PDMS retrieval. The contact angle was measured using an OCA 15EC Drop Shape Analyser–Goniometer (MRG Ibérica, Barcelona, Spain) for each surface.

### 2.3. Biochemical Functionalization of Hydrophilic PMMA Surfaces

Biochemical functionalization of PMMA with cell adhesion proteins was achieved by incubating fibronectin or collagen solutions with the hydrophilic zone of the PMMA surface. Briefly, either 1 µL of fibronectin solution (40 µg mL^−1^) or collagen solution (1 mg mL^−1^) were put on top of the hydrophilic zones and incubated for 1 h. Incubation was carried out in a high-humidity environment to avoid drop evaporation. Afterwards, the drops were removed and the PMMA surface was carefully rinsed with PBS.

For cell adhesion on biochemically functionalized PMMA surfaces, colorectal cancer cells HCT116 were cultured until reaching 80% confluence, detached and re-suspended in culture medium (10% serum) for a final concentration of 2.5 × 10^6^ cells mL^−1^. Drops of 1 µL of cell suspension were put over the hydrophilic zones of the PMMA with fibronectin or collagen functionalization. Drops of 1 µL of cell suspension were also put over the hydrophilic zones without biochemical functionalization.

Simultaneously, PDMS channels (see Supporting Information S1 for specifications) were rinsed with ethanol and adhered to the corresponding PSA film, which was cut to the shape of the PDMS channel using a Graphtec cutting Plotter CE6000-40 (CPS Cutter Printer Systems, Madrid, Spain), covering all areas of the PDMS except the channel.

A cell suspension drop was placed on the functionalized area of the PMMA substrate and the PDMS channel was assembled to the PMMA substrate through the PSA film on top of the pattern. All samples were incubated at 37 °C and 5% CO_2_ inside a cell culture incubator for 1 h in a high-humidity environment to allow cell adhesion to the functionalized PMMA substrate. Afterwards, 200 µL of cell culture medium was flowed three times through hydrostatic pressure to rinse off any of the non-adhered cells. Samples were imaged through brightfield microscopy three times: before rinsing, right after rinsing and 24 h afterwards.

### 2.4. Patterning of Cells in Functionalized PMMA Surfaces

For the patterning of cell adhesion proteins in the hydrophilic surface of PMMA, microcontact printing of fibronectin was carried out. PDMS stamps containing pillars with different features (to generate lollipop-like features, 50 and 100 µm dots) were wetted with 50 µL of a solution of fibronectin 50 µg mL^−1^ and BSA-TAMRA 6.25 µg mL^−1^ for 30 min. Afterwards, the ink was removed and the PDMS stamps were rinsed with DI water and dried over compressed air flow. Each PDMS stamp was put in contact with the hydrophilic zones in the PMMA surface in order to transfer the protein from the PDMS stamp to the substrate and create small dots of fibronectin. Finally, the PDMS stamps were retrieved after 30 min. The same functionalization procedure was carried out in glass cover and polystyrene cell culture wells for comparison.

Then, the patterning of HCT116, prostate cancer cells PC3 and hHF-MSCs was carried out through this procedure. Briefly, cells were cultured until reaching 80% confluence, detached and re-suspended in their respective culture media (10% FBS) for a final concentration of 10^7^ cells mL^−1^. Drops of 1 µL of cell suspension were put over the hydrophilic zones with the fibronectin patterns and incubated at 37 °C and 5% CO_2_ inside a cell culture incubator for 1 h under a high-humidity environment. Afterwards, 200 µL of their corresponding cell culture medium was flown three times through hydrostatic pressure in order to rinse off the non-adhered cells. Brightfield microscopy images were taken after rinsing. The same patterning procedure was carried out for the non-treated PMMA surfaces as a control of protein deposition.

PC3 cells patterned on 50 µm dots were maintained under constant flow (10 µL min^−1^) of medium using a Pump 11 Elite Programmable Syringe Pump (Harvard apparatus, Spain) for 20 h. Brightfield microscopy images were taken afterwards.

For single-cell patterning, PMMA wells were fabricated; see Supporting Information S2 for specifications. PDMS stamps containing arrays of 20 µm dots separated by 50 µm were wetted with 50 µL of a fibronectin solution 200 µg mL^−1^. Later, the ink was removed and the PDMS stamps were rinsed with DI water and dried with compressed air. Each PDMS stamp was put in contact with the PMMA wells for 30 min. The PMMA wells were oxidized as previously explained. After PDMS stamps were retrieved, the wells were loaded with a 2.5% BSA solution in PBS for 15 min. Afterwards, the wells were loaded with 750 µL of either hHF-MSCs or HeLa suspension at a concentration of 20^5^ cells mL^−1^. Half of the samples were left for 30 min on constant oscillation in a rocker inside an incubator at 37 °C and 5% CO_2_ air atmosphere. The other half of the samples was left incubating on a rocker but under static conditions inside an incubator at 37 °C and 5% CO_2_ air atmosphere. Finally, the samples were rinsed three times with PBS.

### 2.5. Imaging and Data Analysis

Brightfield and fluorescence images were taken with a modified Nikon Eclipse TE2000-S (Nikon, Melville, NY, USA) microscope with a LUMENCOR laser light source (Lumencor, Beaverton, OR, USA) and Zylar sCMOS camera (Oxford Instruments, Abingdon, UK). Microscopy images were processed using FiJi/ImageJ software 2.9.0.

## 3. Results and Discussion

### 3.1. Cell Adhesion on Biochemically Functionalized PMMA Surface

Polymethyl methacrylate (PMMA) is a widely used polymer for the fabrication of microfluidic devices, due to its versatility and easy manipulation. While PMMA has moderate biocompatibility, it does not present good properties for cell adhesion. In general, moderate to highly hydrophobic surfaces do not allow the generation of optimal cell–surface interactions, which leads to a lack of important signals required for cell adhesion, proliferation and survival. Therefore, to achieve cell patterning on PMMA, a combined physicochemical and biochemical functionalization of the surface was carried out. Firstly, PMMA was selectively oxidized by air plasma in order to improve its hydrophilicity. Secondly, PMMA surfaces were homogeneously coated with cell adhesion proteins to test the best conditions for cell adhesion. Finally, the cell patterning performance was investigated.

It is known that plasma treatment on PMMA surfaces increases the hydrophilicity of the material and improves cell adhesion [18,19]. By using a PDMS slab with holes as a stencil for the PMMA surface, it is possible to control the plasma exposure of specific areas on the surface of the PMMA. This allows the generation of discrete hydrophilic zones on the surface, which not only improve cell adhesion and protein interaction with the substrate, but also ensure the control of the distribution of cell adhesion zones within the PMMA surface.

**Figure 2:**
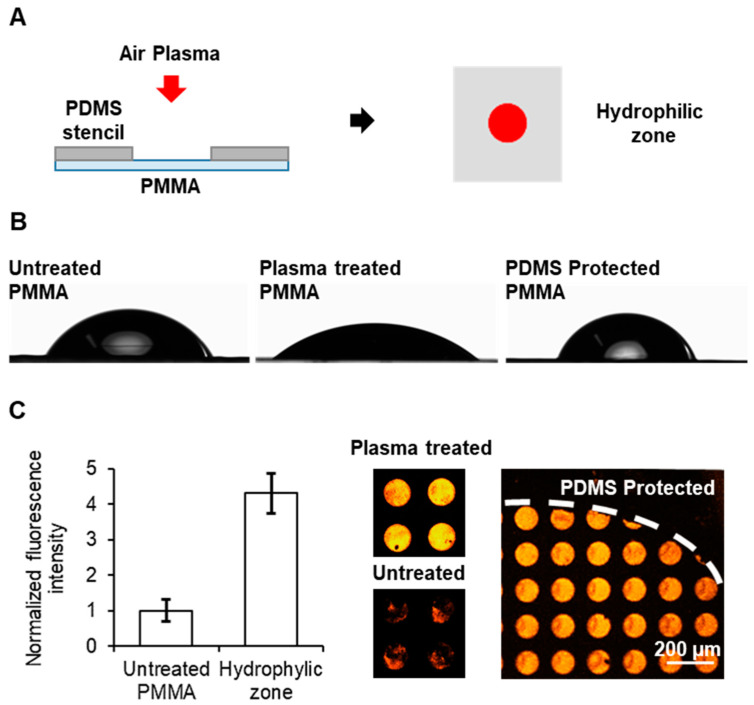
Localized oxidation of PMMA: (**A**) Drawing of the process for localized oxidation of a single zone of the PMMA surface to generate a discrete hydrophilic zone, red spot. (**B**) Photographs for contact angle measurements of drops on PMMA surfaces untreated (**left**), after plasma treatment (**center**) and plasma treated with a PDMS layer on top during the plasma exposition (**right**) for contact angle measurements. (**C**) Plot of the normalized fluorescence intensity (**left**) and fluorescence images (**right**) of PMMA substrates with and without plasma treatment after micropatterning of BSA-TAMRA protein. Error bars mean ± SD (n = 3 samples).

Here, a 3 mm zone of the PMMA surface was treated with air plasma as a way to generate one unique small zone for cell adhesion in a PMMA slide, which would later serve as the cell adhesion zone in a microfluidic device. To generate the 3 mm zone, a PDMS slab with a 3 mm hole was used (Figure 2A). The contact angles of untreated PMMA, plasma-treated PMMA and PDMS-protected PMMA surfaces were measured. As expected, significant differences were observed between the untreated and the fully plasma-treated PMMA surfaces, as the deposited drop spread over the surface, decreasing the value of the contact angle in the treated surface from 62° to 40° (Figure 2B). This confirmed that plasma treatment increased the hydrophilicity and wettability of the treated PMMA surface. Regarding the protected surface, the contact angle increased when compared to the untreated surface, which implies that PDMS protection not only shields the surface from oxidation, but also enhances its hydrophobicity due to the contact and interaction with the PDMS.

For the deposition of fibronectin, microcontact printing (µCP) was tested on the hydrophilic zone the non-treated zones of the PMMA surfaces. Fluorescent BSA-TAMRA was used as reporter of the deposition of the protein. As observed in Figure 2C, a more homogeneous deposition of the protein could be seen in the oxidized zone when compared with non-treated PMMA. Furthermore, a greater deposition of fibronectin was observed on the hydrophilic zone than on the surface that was protected with the PDMS. This confirmed that the transfer of the cell adhesion protein to the PMMA surface was highly dependent on the surface hydrophilicity, which was successfully achieved via controlled air plasma treatment.

In order to enable cell adhesion, the hydrophilic zone was biochemically functionalized with either fibronectin or collagen. Collagen is the main structural protein of the extracellular matrix and promotes cell adhesion to substrates [26]. Fibronectin is another extracellular matrix protein and plays a major role in cell adhesion through the generation of focal adhesions [27]. Adhesion of colorectal cancer cells HCT116 to the hydrophilic zone with and without biochemical functionalization was investigated. After drop deposition, a PDMS channel was assembled onto all PMMA slides. This enabled the development of a straightforward microfluidic device that incorporated a droplet of cell suspension and a hydrophilic zone in the center of the channel. After incubation, culture medium was flowed through the channel to rinse off any non-adhered cells.

**Figure 3:**
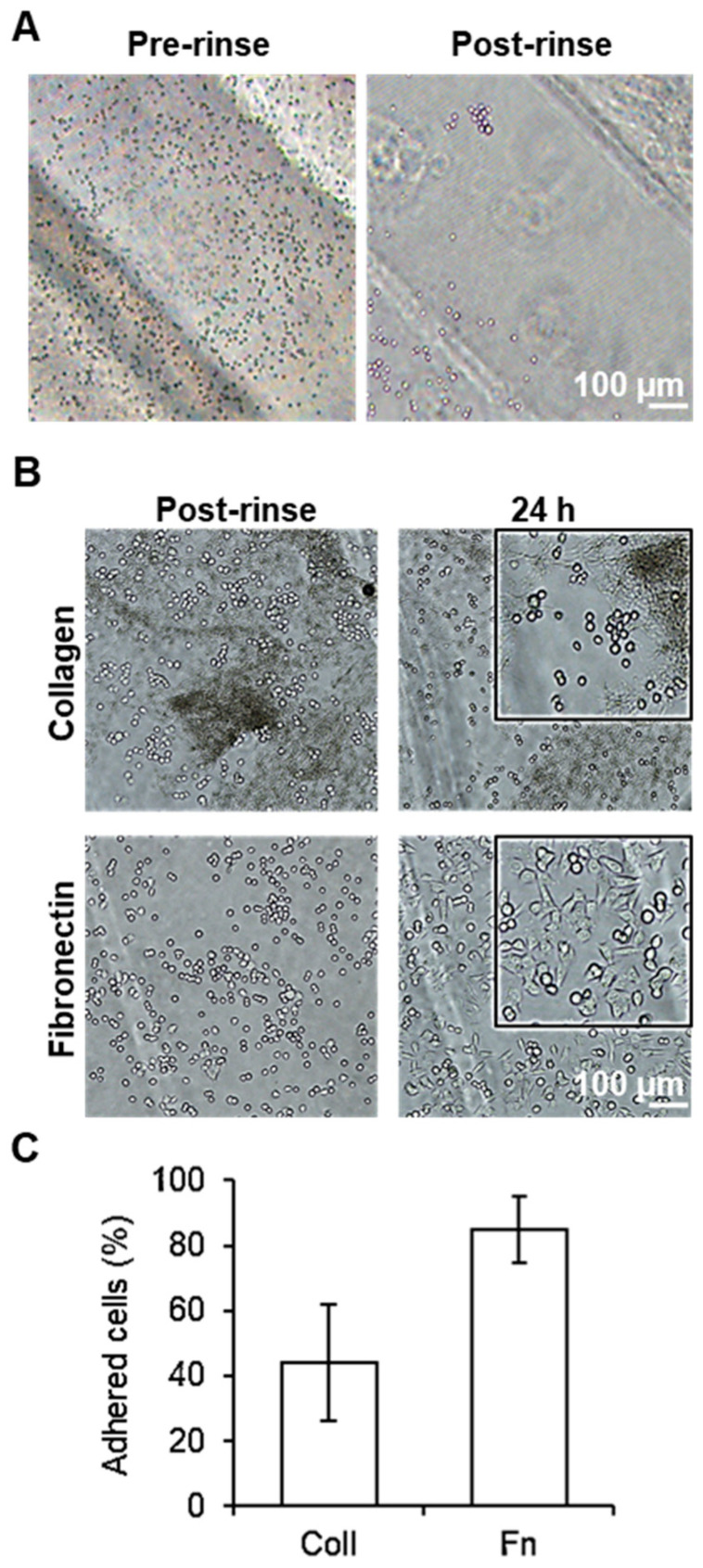
Adhesion of HCT116 cells on PMMA-treated surface: (**A**) Brightfield microscopy images of HCT116 cells cultured before flow (**left**) and after flow (**right**). (**B**) Brightfield microscopy images of cells adhered to collagen (**top row**) and fibronectin (**bottom row**) after flow (**left column**) and 24 h afterwards (**right column**). (**C**) Plot of the percentage of cells adhered to collagen (Coll)- and fibronectin (Fn)-treated hydrophilic zones in the PMMA after rinsing. Error bars mean ± SD (n = 3 samples per experimental condition).

In the case of the samples that did not undergo any biochemical functionalization, the majority of the cells were observed to flow out after rinsing, indicating that the treatment with plasma alone did not induce the formation of focal adhesion points necessary for proper cell adhesion dynamics, see Figure 3A. In contrast, samples treated with either collagen or fibronectin exhibited cell adhesion after only 1 h of incubation. In the case of the collagen samples, approximately 1000 cells could be observed in the substrate after rinsing, indicating that 45% of the cells incubated successfully attached to the PMMA. However, 24 h after the incubation, the number of attached cells decreased by 25%, with the remaining cells displaying a rounded morphology and a lack of spreading, indicating that the cells were not producing optimal interactions with the substrate (Figure 3B,C). In the samples treated with fibronectin, approximately 2100 cells could be found successfully attached to the substrate, corresponding to 85% of the total number of cells incubated. In the case of samples incubated with fibronectin after 24 h, the number of cells on the surface increased to approximately 2200 cells, and presented a more elongated shape, related to a better interaction and adhesion of the cells with the PMMA. This observation suggests that biochemical functionalization with these proteins, combined with the hydrophilic zones generated by the plasma treatment, is a key factor in providing the necessary cues for the adhesion and growth of cells on the PMMA surface.

Out of the two proteins tested, fibronectin presented better results than collagen, as a higher number of cells adhered under the same conditions, and cell proliferation could be observed over the course of 24 h. For such reasons, fibronectin was chosen as the cell adhesion protein for further experiments.

### 3.2. Patterning of Cells on the Hydrophilic PMMA Surfaces

**Figure 4:**
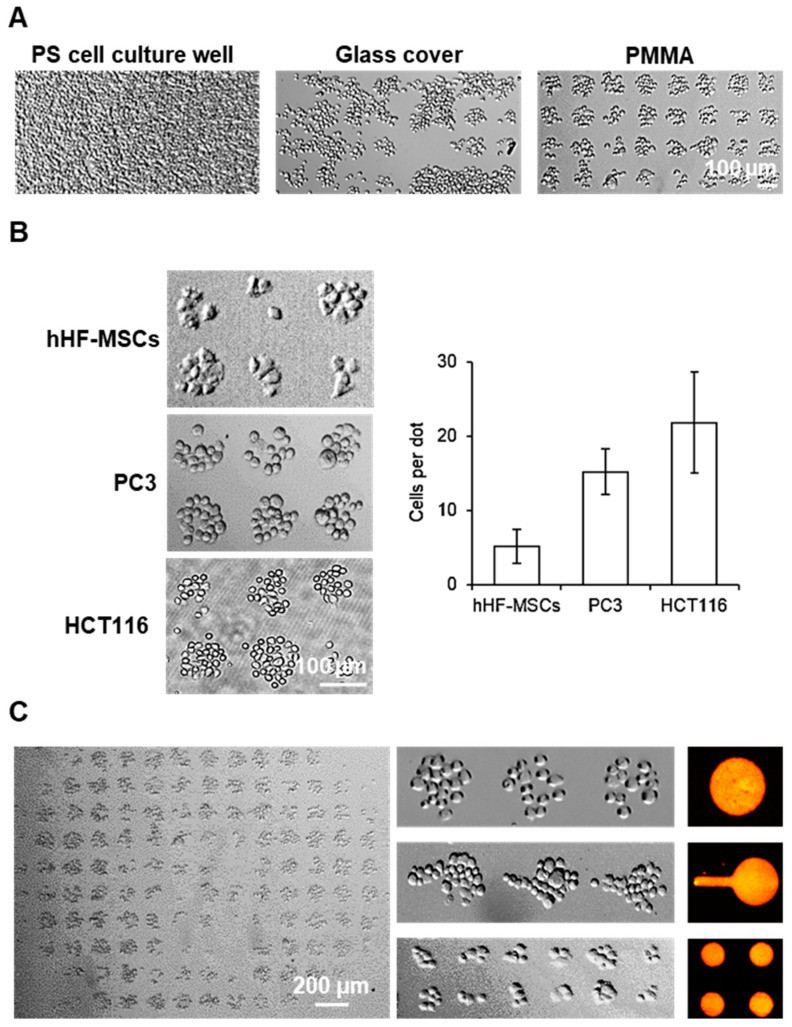
Patterning of cells on PMMA: (**A**) Brightfield images of PC3 patterns on 100 µm dots array of fibronectin with prior hydrophilic zone generation on cell cultures: polystyrene (PS) wells (**top**), glass cover (**middle**) and PMMA surface (**bottom**). (**B**) Brightfield images of the arrays of small cell colonies for hHF-MSCs, PC3 and HCT116 (**top**) and plot of the number of cells per spot (**bottom**). Error bars represent mean ± SD (n = 3 samples per experimental condition). (**C**) Brightfield microscopy images of a full PC3 array on 100 µm fibronectin dot patterns (**left**), pattern of PC3 on 100 µm dots, lollipop-like features and 50 µm dots (**middle column**, **top** to **bottom**) and fluorescence images of fluorescent BSA printed using the same features (**right column**).

Once the improved cell adhesion properties of PMMA after selective plasma treatment and fibronectin coating were demonstrated, the next objective was to achieve controlled cell adhesion in the form of patterns on the PMMA substrate. An array of 100 µm fibronectin dots was produced through µCP on PMMA and subsequently incubated with cells for 1 h. Using brightfield microscopy, patterns of cells were observed on the substrates (Figure 4A). The cytophobic nature of untreated PMMA allowed for highly specific patterning of prostate cancer (PC3) cells on the substrate without the need for any blocking agents. For comparison purposes, the same experiment was performed using glass and conventional polystyrene cell culture well plates. Normally, in order to achieve cell patterns on conventional (hydrophilic) substrates, it is necessary to block non-printed areas with a blocking agent, such as bovine serum albumin, as the absence of a blocking agent results in an unspecific adhesion of cells on conventional cell culture substrates. Contrary to PMMA, the incubation of the glass and polystyrene patterned substrates with the PC3 cells resulted in an expected non-specific cell binding to each substrate, without creating the desired pre-defined patterns. The hydrophilic cell adhesion areas on the PMMA surface contrasted sharply with the surrounding hydrophobic regions, effectively suppressing cell adhesion and migration outside of the pattern. The absence of a blocking step in the cell patterning process on PMMA substrates presents a significant advantage over conventional cell culture substrates, as it eliminates the need for additional steps and reduces the overall time required for the method. This reduction in method time is significant, as it simplifies the process and allows for more efficient cell patterning.

To evaluate the versatility of this simplified methodology to create cell patterns on PMMA substrates, the patterning of three different cell types on PMMA was evaluated, including the previously mentioned HCT116 cells, PC3 cells as well as human mesenchymal stem cells (hHF-MSCs) (Figure 4B). Patterns of small cell colonies for all three types of cells were successfully obtained in all PMMA substrates, generating dozens of individual cell events in a single sample, which can be simultaneously monitored. This confirmed the possibility to control the cell adhesion and cell localization of different adherent cell types through simple PMMA surface manipulation and micropatterning. The number of cells per spot was directly correlated with the size of the cell type itself, as the cells attach and expand as much as the fibronectin spot allows them, where 5 ± 2, 15 ± 3 and 22 ± 6 cells could be found per spot for the patterns with hHF-MSCs, PC3 and HCT116, respectively. This assay implied that this patterning methodology is, in principle, suitable for different cell types and can be adapted to a wide range of scenarios. An analysis of patterned cell viability, Supporting Information S3, showcased that 99% of the patterned cells were alive, confirming that the patterning protocol does not affect cell viability and the suitability of the patterned cells to be directly assayed.

Finally, the possibility of producing cell patterns using different patterns’ motifs was evaluated. Different fibronectin patterns, including 50 µm dots, 100 µm dots and lollipop-like features, were printed on PMMA slides and incubated with PC3, see Supporting Information S4. For all three types of fibronectin arrays studied, the corresponding array of PC3 was obtained, shown in Figure 4C, in which 95% of the dots were occupied. The successful generation of corresponding arrays of PC3 demonstrates for the first time the methodology’s ability to precisely control cell localization and cell–cell interactions through the generation of a different dot shapes and architectures.

The possibility to avoid the blocking step allows the production and fabrication process of the platform to be sped up (from 3.5 to 1.5 h) and further enhances the potential for industrial-scale production of these microtechnologies. Furthermore, the incubation time required to generate the cell patterns has been reduced significantly from the previous reports on PMMA surfaces (from 4 h to 24 h down to 30–60 min [5,24]).

Our methodology presents a straightforward approach for creating cell monitoring devices using PMMA, suitable for a broad spectrum of rapid analytical cell studies. In contrast to traditional cell structures on glass substrates, utilizing PMMA as a foundational substrate offers the advantage of being cost-effective and robust, with adaptability to various fabrication techniques. This choice facilitates the potential for large-scale production and the commercial viability of these platforms. The optical properties of the described patterns, as previously delineated, confer valuable benefits to end-users, as these assays only necessitate optical components for readout. Moreover, the versatility inherent in the types of patterns and cells that can be employed adds to the range of potential applications. These include cell sorting, especially for circulating cells and corpuscles, the assessment of cellular interactions with substrates and biochemical compounds, as well as evaluating cellular responses to chemical stimuli. Our previous works on the use of cell patterns for the optical monitoring of cell behaviors (monitoring of cell adhesion and biosensing of their integrin profile, cell death under cytotoxic treatments and cell transfection under controlled cell–cell contact) would benefit from these advantages to evolve into fully formed devices with manufacturing and commercialization prospects [4,28,29].

### 3.3. Maintenance of the Cell Patterns under Flow Conditions

Mechanical forces related to fluid flow, such as shear stress, have been shown to significantly impact cellular behavior under physiological conditions, altering key cellular processes such as attachment dynamics, proliferation rates and gene expression, among others [30,31,32]. For such reasons, there is an increasing interest in the development of microfluidic devices, which allows for the studying and monitoring of cell patterns under controlled flow conditions.

**Figure 5:**
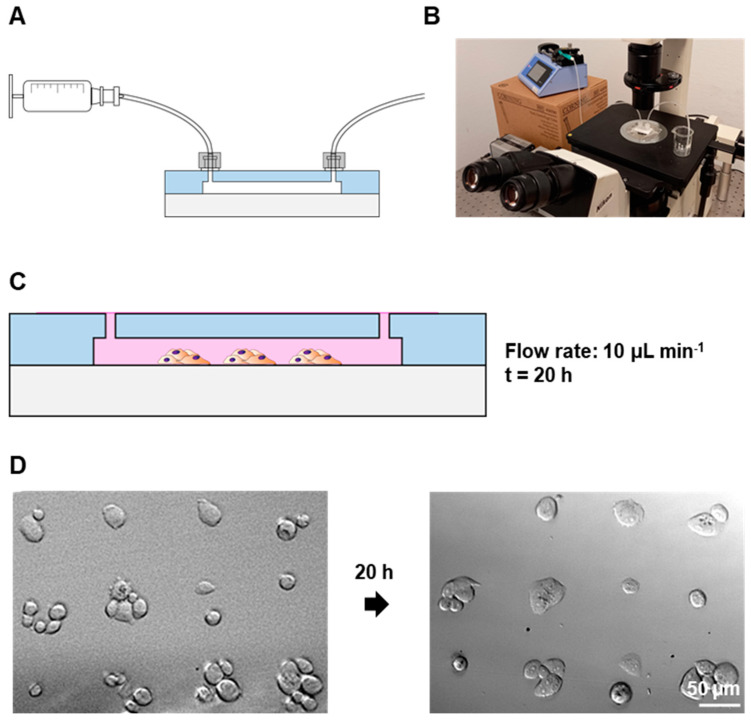
Maintenance of cell patterns under flow conditions: (**A**) Schematic drawing of the syringe–device connection. (**B**) Photograph of the setup. (**C**) Schematic drawing of patterned cell maintenance inside the chip. (**D**) Brightfield microscopy images of PC3 cells on 50 µm patterns after patterning and after 20 h on flow (10 µL min^−1^).

The PC3 patterns in the 50 µm fibronectin dots were maintained in a constant flow of 10 µL min^−1^ overnight; see Figure 5A,B for setup specifications. While some cells detached during the process, the cells were still confined in their respective dots and the patterns remained similar to their original state for at least 20 h; see Figure 5C,D.

These findings suggest, for the first time, that the cell arrays on PMMA surfaces can be readily integrated and analyzed within a microfluidic device under precisely controlled flow conditions, thereby conferring practical applicability to this platform. The advancement of PMMA-based microfluidic devices holds considerable promise for end-users. These devices can be rapidly manufactured and scaled up efficiently. Optical monitoring further attests to the simplicity of operation, negating the need for intricate instrumentation. These microfluidic platforms can be utilized for the swift and quick analysis of cell adhesion, detachment and viability under meticulously controlled chemical and fluidic conditions.

It should be noted that both cell adhesion and the overnight incubation were performed using a cell culture medium supplemented with serum. The growth and migration factors contained in the serum may cause the cells to move and proliferate outside of the established pattern when the method is performed in conventional cell culture substrates. The absence of unspecific attachment or cell migration outside of the pattern in the PMMA samples is attributed to the robust cytophobic nature of the non-treated PMMA substrate, which effectively restrains cells from moving or attaching outside of the designated cell adhesion spots. This feature of PMMA provides an additional benefit over conventional cell culture materials.

### 3.4. Single-Cell Patterning on the Hydrophilic PMMA Surfaces

As the incubation of cell suspension with the hydrophilic zone in static conditions can lead to the formation of cell–cell interactions, resulting in stronger bonds compared to those formed with the fibronectin dot, a modified µCP protocol was utilized for generating single-cell patterns.

PMMA wells were fabricated and 20 µm fibronectin arrays were produced through µCP on the oxidized PMMA surface. The patterns were incubated with a BSA solution for 15 min in order to avoid an accumulation of cell clusters within the surface, which could have led to inefficient interactions between the cells and the tiny fibronectin dots. The previously mentioned hHF-MSCs and HeLa cells were incubated with the fibronectin arrays, both on constant oscillation and in static conditions.

**Figure 6:**
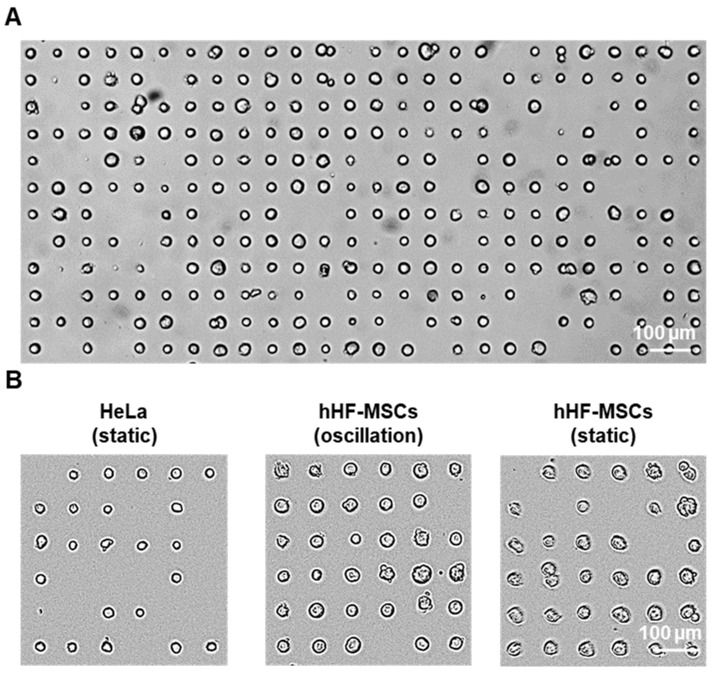
Single-cell patterning on PMMA: (**A**) Brightfield microscopy image of >200 single hHF-MSCs patterned in oscillation conditions. (**B**) Brightfield microscopy images of HeLa and hHF-MSCs patterns on 20 µm patterns after 30 min incubation.

As seen in Figure 6, single-cell adhesion of both cell types was obtained. In the case of the MSCs, the majority of the fibronectin dots were occupied by cells after incubating for just 30 min (75% ± 4 and 80% ± 6 for the patterns incubated in static and oscillation conditions, respectively). In most cases, only one cell adhered to the fibronectin dots (95% ± 3 and 97.7% ± 0.5 for the patterns incubated in static and oscillation conditions, respectively), and the patterns presented almost no unspecific adhesion of cells on the PMMA surface. In the case of the HeLa cells incubated in static conditions, 57% ± 10 of the fibronectin dots were occupied after a 30 min incubation of the cell suspension with the pattern. HeLa cells incubated under constant oscillation displayed no cell adhesion due to the high agglomeration and clustering of the cells in suspension. Similarly to the single-MSC patterns, only one cell adhered to most of the fibronectin dots (95% ± 4) and almost no unspecific adhesion of cells on the PMMA surface could be observed.

These findings provide additional evidence to support the viability of our approach to enable and regulate cell adhesion, and demonstrate the potential of the method for use in single-cell monitoring and biosensing applications. Although a blocking agent was necessary to produce this form of cell array, the time required for the blocking process was reduced by at least a quarter compared to earlier reports on single-cell patterning, due in part to the intrinsic cytophobic properties of PMMA previously discussed. The differences that could be observed between different cell types and incubation conditions are expected, as the specific protocol should be optimized for each cell type and assay. Thanks to the great versatility of the method, several parameters (including the dimensions of the adhesion features, the cell adhesion proteins used, the concentration of cells, the time of incubation and the presence or absence of motion during cell adhesion) can be altered in order to achieve the optimal conditions for each type of bioassay. These findings have the potential to significantly influence our previously established single-cell adhesion dot array (SCADA) methodology. This innovative technique facilitates optical assessment of the variables affecting cellular adhesion and detachment without requiring complex instrumentation. Consequently, it offers a pathway to developing user-friendly microfluidic devices for the convenient monitoring of cellular behaviors [4,28].

## 4. Conclusions

In this study, we investigated a novel approach for achieving controlled cell adhesion and patterning on PMMA, a widely employed polymer in the fabrication of microfluidic devices that has not been extensively utilized in commercial cell monitoring microtechnologies due to its inherent cytophobicity. PMMA surfaces were physicochemical modified with localized air plasma in order to improve their hydrophilicity, while microcontact printing was used for the patterning of different cell types with controlled cell localization and cell–cell contact. The exploration of PMMA surface functionalization and cell–material interactions has resulted in an improvement in the polymer’s cell adhesion capabilities. This advancement not only transformed PMMA from a cytophobic to a cytophilic material, but also allowed for the creation of precise patterns of various cell types. The ease of manipulation and handling of PMMA, along with its significantly reduced production time for cell arrays and direct integration of patterns into microfluidic devices, make this method user-friendly and suitable for both academic research and industrial mass-production processes. Overall, this work will contribute to the development of commercial products aimed at cell-based biosensing and the monitoring of cell behavior. It can be combined with numerous microfabrication-based methods that are being developed for the pharmaceutical industry and biological research laboratories worldwide, thus further expanding its potential applications.

## Data Availability

Data is available from the authors upon request.

## Supplementary Materials

The following supporting information [4] can be downloaded at: https://www.mdpi.com/article/10.3390/bios13100904/s1, Figure S1: PDMS channel specifications; Figure S2: PMMA wells specifications; Figure S3: Viability assay of cell patterns on PMMA; Figure S4: Printed fibronectin features.

## References

[1] Streets A.M., Huang Y. (2013). Chip in a lab: Microfluidics for next generation life science research. Biomicrofluidics.

[2] Azuaje-Hualde E., García-Hernando M., Etxebarria-Elezgarai J., De Pancorbo M., Benito-Lopez F., Basabe-Desmonts L. (2017). Microtechnologies for Cell Microenvironment Control and Monitoring. Micromachines.

[3] Coluccio M.L., Perozziello G., Malara N., Parrotta E., Zhang P., Gentile F., Limongi T., Raj P.M., Cuda G., Candeloro P. (2019). Microfluidic platforms for cell cultures and investigations. Microelectron. Eng..

[4] Garcia-Hernando M., Calatayud-Sanchez A., Etxebarria-Elezgarai J., de Pancorbo M.M., Benito-Lopez F., Basabe-Desmonts L. (2020). Optical Single Cell Resolution Cytotoxicity Biosensor Based on Single Cell Adhesion Dot Arrays. Anal. Chem..

[5] Hager R., Forsich C., Duchoslav J., Burgstaller C., Stifter D., Weghuber J., Lanzerstorfer P. (2022). Microcontact Printing of Biomolecules on Various Polymeric Substrates: Limitations and Applicability for Fluorescence Microscopy and Subcellular Micropatterning Assays. ACS Appl. Polym. Mater..

[6] Matellan C., del Río Hernández A.E. (2018). Cost-effective rapid prototyping and assembly of poly(methyl methacrylate) microfluidic devices. Sci. Rep..

[7] Liga A., Morton J.A.S., Kersaudy-Kerhoas M. (2016). Safe and cost-effective rapid-prototyping of multilayer PMMA microfluidic devices. Microfluid. Nanofluid..

[8] Rega R., Gennari O., Mecozzi L., Pagliarulo V., Mugnano M., Oleandro E., Nazzaro F., Ferraro P., Grilli S. (2019). Pyro-Electrification of Freestanding Polymer Sheets: A New Tool for Cation-Free Manipulation of Cell Adhesion in vitro. Front. Chem..

[9] Mecozzi L., Gennari O., Rega R., Grilli S., Bhowmick S., Gioffrè M.A., Coppola G., Ferraro P. (2016). Spiral formation at the microscale by μ-pyro-electrospinning. Soft Matter.

[10] Sharifi R., Mahmoudzadeh S., Islam M.M., Koza D., Dohlman C.H., Chodosh J., Gonzalez-Andrades M. (2020). Covalent Functionalization of PMMA Surface with L-3,4-Dihydroxyphenylalanine (L-DOPA) to Enhance its Biocompatibility and Adhesion to Corneal Tissue. Adv. Mater. Interfaces.

[11] Jaganjac M., Milković L., Cipak A., Mozetič M., Recek N., Žarković N., Vesel A. (2012). Cell Adhesion On Hydrophobic Polymer Surfaces. Mater. Tehnol..

[12] Cai S., Wu C., Yang W., Liang W., Yu H., Liu L. (2020). Recent advance in surface modification for regulating cell adhesion and behaviors. Nanotechnol. Rev..

[13] Riau A.K., Venkatraman S.S., Mehta J.S. (2020). Biomimetic vs. Direct Approach to Deposit Hydroxyapatite on the Surface of Low Melting Point Polymers for Tissue Engineering. Nanomaterials.

[14] Apostol M., Mironava T., Yang N.-L., Pernodet N., Rafailovich M.H. (2011). Cell sheet patterning using photo-cleavable polymers. Polym. J..

[15] Riau A.K., Mondal D., Yam G.H.F., Setiawan M., Liedberg B., Venkatraman S.S., Mehta J.S. (2015). Surface Modification of PMMA to Improve Adhesion to Corneal Substitutes in a Synthetic Core–Skirt Keratoprosthesis. ACS Appl. Mater. Interfaces.

[16] Patel S., Thakar R.G., Wong J., McLeod S.D., Li S. (2006). Control of cell adhesion on poly(methyl methacrylate). Biomaterials.

[17] Welle A., Gottwald E. (2002). UV-Based patterning of polymeric substrates for cell culture applications. Biomed. Microdevices.

[18] Kanioura A., Constantoudis V., Petrou P., Kletsas D., Tserepi A., Gogolides E., Chatzichristidi M., Kakabakos S. (2020). Oxygen plasma micro-nanostructured PMMA plates and microfluidics for increased adhesion and proliferation of cancer versus normal cells: The role of surface roughness and disorder. Micro Nano Eng..

[19] Detrait E., Lhoest J.-B., Knoops B., Bertrand P., van den Bosch de Aguilar P. (1998). Orientation of cell adhesion and growth on patterned heterogeneous polystyrene surface. J. Neurosci. Methods.

[20] Kanioura A., Petrou P., Kletsas D., Tserepi A., Chatzichristidi M., Gogolides E., Kakabakos S. (2020). Three-dimensional (3D) hierarchical oxygen plasma micro/nanostructured polymeric substrates for selective enrichment of cancer cells from mixtures with normal ones. Colloids Surf. B Biointerfaces.

[21] Bhujbal S.V., Dekov M., Ottesen V., Dunker K., Lale R., Sletmoen M. (2020). Effect of design geometry, exposure energy, cytophilic molecules, cell type and load in fabrication of single-cell arrays using micro-contact printing. Sci. Rep..

[22] Delamarche E., Pereiro I., Kashyap A., Kaigala G.V. (2021). Biopatterning: The Art of Patterning Biomolecules on Surfaces. Langmuir.

[23] Schmalenberg K.E., Uhrich K.E. (2005). Micropatterned polymer substrates control alignment of proliferating Schwann cells to direct neuronal regeneration. Biomaterials.

[24] Schmalenberg K.E., Buettner H.M., Uhrich K.E. (2004). Microcontact printing of proteins on oxygen plasma-activated poly(methyl methacrylate). Biomaterials.

[25] Wang D.-Y., Huang Y.-C., Chiang H., Wo A.M., Huang Y.-Y. (2007). Microcontact printing of laminin on oxygen plasma activated substrates for the alignment and growth of Schwann cells. J. Biomed. Mater. Res. Part B Appl. Biomater..

[26] Heino J. (2007). The collagen family members as cell adhesion proteins. Bioessays.

[27] Hsiao C.-T., Cheng H.-W., Huang C.-M., Li H.-R., Ou M.-H., Huang J.-R., Khoo K.-H., Yu H.W., Chen Y.-Q., Wang Y.-K. (2017). Fibronectin in cell adhesion and migration via N-glycosylation. Oncotarget.

[28] Gonzalez-Pujana A., Santos-Vizcaino E., García-Hernando M., Hernaez-Estrada B., de Pancorbo M.M., Benito-Lopez F., Igartua M., Basabe-Desmonts L., Hernandez R.M. (2019). Extracellular matrix protein microarray-based biosensor with single cell resolution: Integrin profiling and characterization of cell-biomaterial interactions. Sens. Actuators B Chem..

[29] Azuaje-Hualde E., Rosique M., Calatayud-Sanchez A., Benito-Lopez F., de Pancorbo M.M., Basabe-Desmonts L. (2021). Continuous monitoring of cell transfection efficiency with micropatterned substrates. Biotechnol. Bioeng..

[30] Zhang X., Jones P., Haswell S. (2008). Attachment and detachment of living cells on modified microchannel surfaces in a microfluidic-based lab-on-a-chip system. Chem. Eng. J..

[31] Murthy S.K., Radisic M. (2013). Cell Adhesion and Detachment. Encyclopedia of Microfluidics and Nanofluidics.

[32] Salvi J.D., Lim J.Y., Donahue H.J. (2010). Finite Element Analyses of Fluid Flow Conditions in Cell Culture. Tissue Eng. Part C Methods.

