# Supplementary material for: Cell Patterning Technology on Polymethyl Methacrylate through Controlled Physicochemical and Biochemical Functionalization
Source: Biosensors (Basel). 2023 Sep 23;13(10):904. doi: 10.3390/bios13100904 (PMC10604931; doi:10.3390/bios13100904)
Supplement: Supplementary file 1 [file biosensors-13-00904-s001.zip › biosensors-2532316-supplementary.pdf]

Cell Patterning Technology on Polymethyl Methacrylate through Controlled Physicochemical and Biochemical Functionalization

Enrique Azuaje-Hualde [1,2], Job Komen [3], Juncal A. Alonso-Cabrera [1,4], Albert van den Berg [3], Marian M. de Pancorbo [5], Andries D. van der Meer [6], Fernando Benito-Lopez [2,4,*] and Lourdes Basabe-Desmonts [1,2,7,*]

1 Microfluidics Cluster UPV/EHU, BIOMICs Microfluidics Group, Lascaray Research Center, University of the Basque Country UPV/EHU, 01006 Vitoria-Gasteiz, Spain; enrique.azuaje@ehu.eus (E.A.-H.); juncalanne.alonso@ehu.eus (J.A.A.-C.)
2 Bioaraba Health Research Institute, Microfluidics Cluster UPV/EHU, 01009 Vitoria-Gasteiz, Spain
3 BIOS Lab on a Chip Group, MESA+ Institute for Nanotechnology, University of Twente, P.O. Box 217, 7500 AE Enschede, The Netherlands; jobkomen@gmail.com (J.K.); a.vandenberg@utwente.nl (A.v.d.B.)
4 Microfluidics Cluster UPV/EHU, Analytical Microsystems & Materials for Lab-on-a-Chip (AMMa-LOAC) Group, Analytical Chemistry Department, University of the Basque Country UPV/EHU, 48940 Leioa, Spain
5 BIOMICs Research Group, Lascaray Research Center, University of the Basque Country UPV/EHU, 01006 Vitoria-Gasteiz, Spain; marianpancorbo@gmail.com
6 Applied Stem Cell Technologies, TechMed Centre, University of Twente, P.O. Box 217, 7500 AE Enschede, The Netherlands; andries.vandermeer@utwente.nl
7 Basque Foundation of Science, IKERBASQUE, María Díaz Haroko Kalea, 3, 48013 Bilbao, Spain
* Correspondence: fernando.benito@ehu.eus (F.B.-L.); lourdes.basabe@ehu.eus (L.B.-D.); Tel.: +34-94-45-01-45-38 (L.B.-D.)

Supporting Information

S1. PDMS channel specifications

A PDMS channel was fabricated and adhered directly on top to the cell patterns through pressure sensitive adhesive, **Figure S1.**

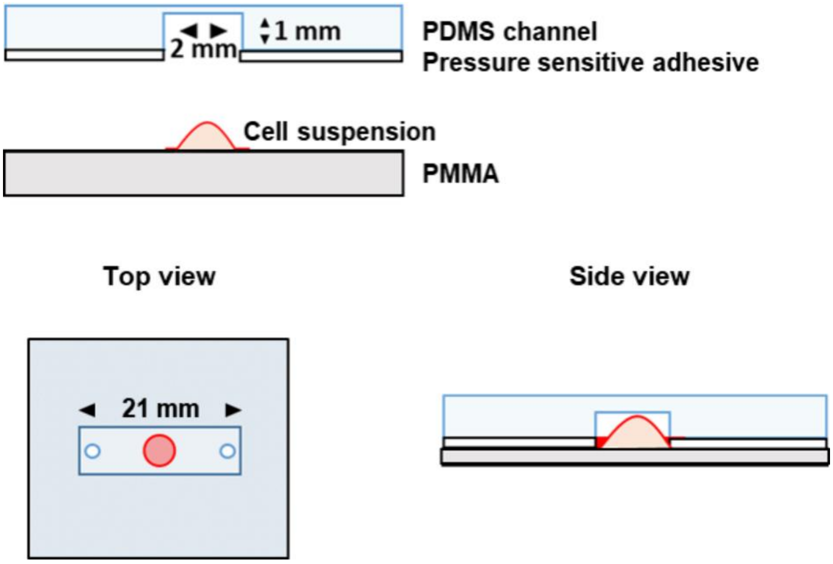

Figure S1. PDMS channel specifications.

S2. PMMA wells specifications

In order to generate wells (2 cm) made of PMMA, three PMMA layers (4 mm) were cut with a laser and pasted both between them and with the glass cover with pressure sensitive adhesive (PSA). **Figure S2**.

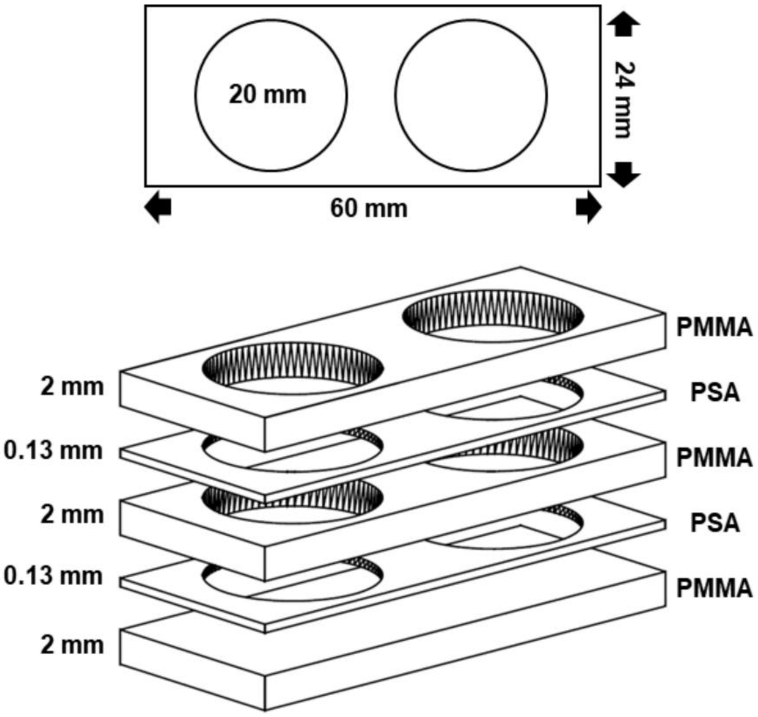

Figure S2. PMMA wells specifications.

S3. Viability assay of cell patterns on PMMA.

Viability of patterned cells was studied directly after patterning (t 0) and after an incubation of 24 h (t 24). For this assay, HeLa cells were patterned in arrays of small cell-isles by printing 100 µm fibronectin dots. Patterning protocol and cell adhesion protocol were followed as described in Section 2.4. in the manuscript.

To assess cell viability, a trypan blue assay for patterned cells was followed as previously described [1]. In short, cell culture medium was retrieved from each well either directly after patterning, or after a 24 h incubation at 37 ºC and 5 % CO_2 inside a cell culture incubator with serum-free DMEM medium. 4 samples were used for each condition. Afterwards, the samples were washed twice with PBS, and 50% (v/v) of trypan blue in DMEM was added to immediately track their viability. Brightfield images and viability assay were performed with a Corning Cell Counter, Fisher Scientific, Spain. Viability was analyzed using Axion Biosystems Portal.

Viability of patterned cells directly after the patterning protocol (t 0) was 99 %, indicating that the vast majority of the patterned cells were alive within the patterns, which in turn confirms that the patterning protocol does not affect negatively the cells' viability. In the patterns that were incubated for 24 h, a loss in the number of cells could be observed (10 ± 2 and 8 ± 3 cells for the patterns at t 0 and t 24, respectively) indicating that between 10 % and 20 % of the patterned cells detach over the course of 24 h. Nevertheless, viability of the patterns at t 24 was still 98.5 %, indicating that the cells that the vast majority of cells that remain in the patterns are alive. Furthermore, cells elongated over the course of the 24 h, which indicate proper adhesion of the cells with the substrate, **Figure S3**.

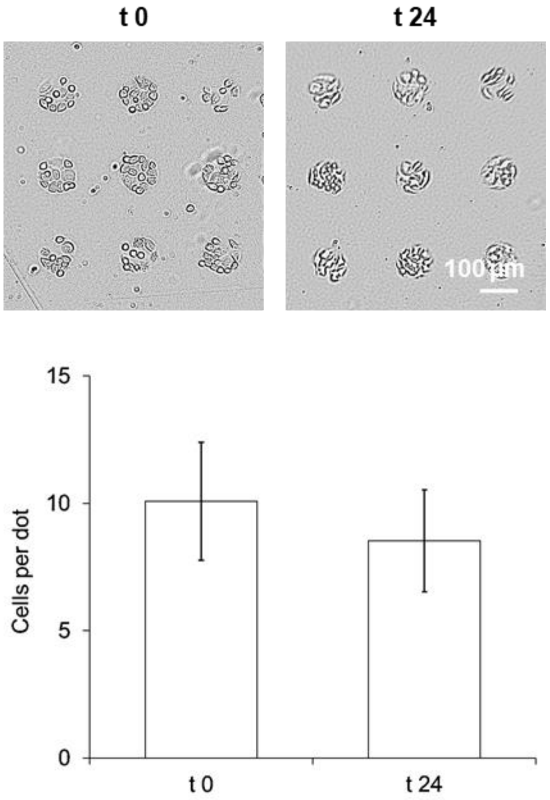

Figure S3. Viability assay of patterned cells. Top; Brightfield images of patterned HeLa cells in 100 µm fibronectin dots after patterning (t 0) and after 24 h incubation (t 24). Bottom; Graphic of the number of cells per dot at t 0 and t 24. Error bars means SD (180 dots measured from 4 samples).

S4. Printed fibronectin features

For the patterning of cells with different cell-cell contact and cell confluences, different arrays of fibronectin features were printed, **Figure S4**.

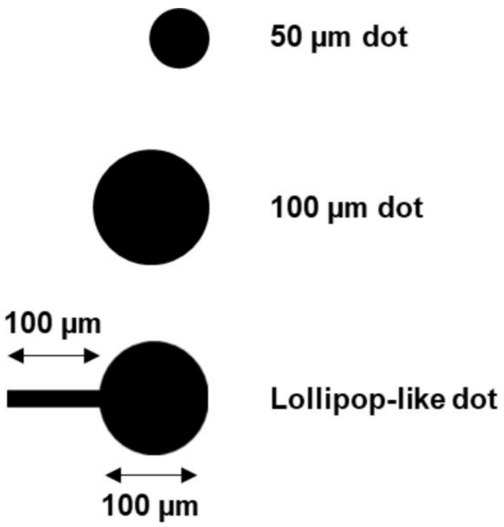

Figure S4. Fibronectin features printed for cell adhesion.

References

[1] Garcia-Hernando, M.; Calatayud-Sanchez, A.; Etxebarria-Elezgarai, J.; de Pancorbo, M.M.; Benito-Lopez, F.; Basabe-Desmonts, L. Optical Single Cell Resolution Cytotoxicity Biosensor Based on Single Cell Adhesion Dot Arrays. *Anal. Chem.* **2020**, *92*, 9658–9665. https://doi.org/10.1021/acs.analchem.0c00940
